# Supplementary material for: Changes in the distribution of the tear film lipid layer after intensive pulsed light combined with meibomian gland expression in patients with meibomian gland dysfunction
Source: PLoS One. 2025 Feb 28;20(2):e0316935. doi: 10.1371/journal.pone.0316935 (PMC11870382; doi:10.1371/journal.pone.0316935)
Supplement: Attachment — Submitted filename: Comments to the Editor .docx [file pone.0316935.s001.docx]

**Friday, January 3**

**Dear Editor**

Thank you for the invitation to review the manuscript titled: **Changes in the distribution of the tear lipid layer after intensive pulse light combined meibomian gland expression in patients with meibomian gland dysfunction**.

The aim of the study is to investigate changes in dry eye disease (DED) parameters and lipid-layer distribution after treatment with intensive pulse light combined with Meibomian gland expression treatment in patients with Meibomian gland dysfunction (MGD). After reviewing the manuscript, I think it requires a major revision to be suitable for publication in your admired journal. However, some comments to the author are presented below to improve the manuscript:

**Materials and Methods**

1. Page 5, lines 39-35: please clear the treatment periods (What is the interval between sessions, and why were the measurements done two weeks after IPL, not earlier?
2. The method should explain the light parameters used during IPL therapy using an acne filter, such as the emitted light wavelength, light intensity, operating voltage, power, frequency, maximum optical energy, pulse duration, and repetition time or any data regarding the instrument used.
3. The author mentioned in the abstract that: This retrospective study included 218 patients diagnosed with MGD, while in the result section (Page 7, line 128), he mentioned the number of patients was 337. In addition, the number of patients should be added obviously in the method section.
4. **In the discussion section**, **pages 10-11**, from lines 182-194, there are obvious overlaps. These paragraphs can be summarized in one or two sentences only.
5. **Page 13, lines 232-236**: there was no relation between the two sentences about the treated eyes and the healthy individuals. Please try to find another reference to support your data.
6. **Page 13, lines 241-242**. The distribution of the lipids layer is affected by blinking as each time the eye blinks, the lipid layer on the surface of the eye is compressed and then steadily expands, which creates a non-equilibrium state, and affects the reorganization of the lipid layers. The author should enrich the discussion with previous studies regarding tear lipids as PMID: 30820280 and other previous studies.
7. The results obtained are not discussed carefully in the discussion section in addition to the weaknesses in the correlation between the obtained results and the previous literature.

Best Regards
